# Rapamycin and Resveratrol Modulate the Gliotic and Pro-Angiogenic Response in Müller Glial Cells Under Hypoxia

**DOI:** 10.3389/fcell.2022.855178

**Published:** 2022-03-01

**Authors:** Paula V. Subirada, María V. Vaglienti, Mariana B. Joray, María C. Paz, Pablo F. Barcelona, María C. Sánchez

**Affiliations:** ^1^ Universidad Nacional de Córdoba, Facultad de Ciencias Químicas, Departamento de Bioquímica Clínica, Córdoba, Argentina; ^2^ Consejo Nacional de Investigaciones Científicas y Técnicas (CONICET), Centro de Investigaciones en Bioquímica Clínica e Inmunología (CIBICI), Córdoba, Argentina; ^3^ Universidad Católica de Córdoba, Facultad de Ciencias Químicas, Córdoba, Argentina; ^4^ Consejo Nacional de Investigaciones Científicas y Técnicas (CONICET), Instituto de Investigaciones en Recursos Naturales y Sustentabilidad José Sánchez Labrador J. S., Córdoba, Argentina

**Keywords:** autophagy, hypoxia, angiogenesis, vascular endothelial growth factor, pigment epithelium-derived factor, gliosis

## Abstract

Hypoxia and hypoxia-reoxygenation are frequently developed through the course of many retinal diseases of different etiologies. Müller glial cells (MGCs), together with microglia and astrocytes, participate firstly in response to the injury and later in the repair of tissue damage. New pharmacological strategies tend to modulate MGCs ability to induce angiogenesis and gliosis in order to accelerate the recovery stage. In this article, we investigated the variation in autophagy flux under hypoxia during 4 h, employing both gas culture chamber (1% O_2_) and chemical (CoCl_2_) hypoxia, and also in hypoxia-reoxygenation. Then, we delineated a strategy to induce autophagy with Rapamycin and Resveratrol and analysed the gliotic and pro-angiogenic response of MGCs under hypoxic conditions. Our results showed an increase in LC3B II and p62 protein levels after both hypoxic exposure respect to normoxia. Moreover, 1 h of reoxygenation after gas hypoxia upregulated LC3B II levels respect to hypoxia although a decreased cell survival was observed. Exposure to low oxygen levels increased the protein expression of the glial fibrillary acid protein (GFAP) in MGCs, whereas Vimentin levels remained constant. In our experimental conditions, Rapamycin but not Resveratrol decreased GFAP protein levels in hypoxia. Finally, supernatants of MGCs incubated in hypoxic conditions and in presence of the autophagy inductors inhibited endothelial cells (ECs) tubulogenesis. In agreement with these results, reduced expression of vascular endothelial growth factor (VEGF) mRNA was observed in MGCs with Rapamycin, whereas pigment epithelium-derived factor (PEDF) mRNA levels significantly increased in MGCs incubated with Resveratrol. In conclusion, this research provides evidence about the variation of autophagy flux under hypoxia and hypoxia-reoxygenation as a protective mechanism activated in response to the injury. In addition, beneficial effects were observed with Rapamycin treatment as it decreased the gliotic response and prevented the development of newly formed blood vessels.

## Introduction

As part of the Central Nervous System, the retina is highly dependent on nutrients and bioactive molecules, among them oxygen (Country, 2017). Extensive research has demonstrated that excessive or defective supply of oxygen is sufficient to trigger retinal damage or dysfunction (Caprara and Grimm, 2012). Retinopathy of prematurity (ROP) is a neonatal pathology that evidence this statement, as the events taking place in both the hyperoxic and the hypoxic phases are responsible for neuronal demise and glial activation (Ridano et al., 2017). In other retinal disorders as retinal vein occlusion, sickle cell anemia or diabetic retinopathy during the proliferative stage (PDR), hypoxia is solely detected.

Either constant or intermittent hypoxia is perceived as a threatening stimulus for retinal neurons. As a consequence, a fast response is settled by glial cells. Müller glial cells (MGCs), the most abundant macroglial cells of the retina, are known to collaborate in the immune response by secreting several cytokines, and to lead the vaso-proliferative response under hypoxia (Liu et al., 2014; Eastlake et al., 2016; Li et al., 2019). Many proteins participate in angiogenesis, including pro-angiogenic factors as the vascular endothelial growth factor (VEGF) and anti-angiogenic factors as pigment epithelium-derived factor (PEDF) (Eichler et al., 2004). Although these factors are secreted by many retinal cells, conditional VEGF KO mice revealed that the contribution of MGCs is indispensable for the vascular proliferation (Wang et al., 2010). Several signaling pathways can regulate VEGF synthesis or secretion. Therefore, a wide variety of compounds are able to modulate VEGF during pathological angiogenesis (Formica et al., 2021; Szymanska et al., 2021).

Another protective mechanism activated upon damage is autophagy. Autophagy is an intracellular catabolic process that ensure the degradation of misfolded proteins and altered organelles in the damaged tissue. Changes in autophagic flux can either induce cell survival or death. In this sense, treatments with different autophagy modulators proved to reduce cellular death or improve tissue function in disease (Kunchithapautham et al., 2011; Lin and Xu, 2019). On the other hand, autophagy inductors have demonstrated to exert pleiotropic effects in cells, including the secretion of trophic factors, affecting the fate of neighbor cells (Li et al., 2014; Zhou et al., 2018; Zwanzig et al., 2021). Two potent autophagy inductors are Rapamycin and Resveratrol. These compounds have attracted increasing attention in the research field because of their antioxidant and anti-angiogenic effects (Lee et al., 2015; Xin et al., 2017; Zhou et al., 2021). In ischemic retinopathies, it results interesting to evaluate if autophagy inductors under hypoxic conditions modulate the secretion of anti- or pro-angiogenic factors in MGCs and therefore vascular outgrowth.

Here, we initially investigated the variation in the autophagy flux under hypoxia and hypoxia-reoxygenation. Based on these results, we then delineated a strategy to induce autophagy with Rapamycin and Resveratrol and analysed the gliotic and pro-angiogenic response of MGCs under hypoxic conditions.

## Materials and Methods

### Cell Line and Culture Reagents

A spontaneously immortalized human MGC line (MIO-M1), kindly provided by G. Astrid Limb (UCL Institute of Ophthalmology and Moorfields Eye Hospital, London, United Kingdom), was used. Cells were grown in Dulbecco’s modified Eagle’s medium (DMEM; Invitrogen, Buenos Aires, Argentina) containing 4500 mg/L glucose, sodium pyruvate) with 2 mM L-glutamine (GlutaMAX; Invitrogen), 10% vol/vol fetal bovine serum (FBS), and 50 U/mL penicillin/streptomycin (Invitrogen). Bovine aortic endothelial cells (BAECs) were grown in DMEM supplemented with 20% vol/vol FBS, and penicillin (100 U/mL)/streptomycin (100 U/mL) (Invitrogen). BAECs’ media contained 0, 03 mg/ml of endothelial cell growth supplement from bovine pituitary (Sigma). Cell cultures were kept at 37°C in a 5% CO_2_ humidified environment.

### Hypoxic Assays

For gas hypoxia, cells were grown at 50–60% confluence in normal conditions and then transferred to a gas culture chamber (StemCell Technologies, Vancouver, Canada) supplied with 1% O_2_, 94% N_2_, and 5% CO_2_. Control cells were kept in normoxia (21% O_2_). For chemical hypoxia, cells were grown at 50–60% confluence in 10% FBS and then a solution of CoCl_2_ (Cicarelli, Santa Fe, Argentina) diluted in PBS was added to the cell medium. Cell experiments were conducted at two different time points (4 and 24 h). To assess the autophagy flux, Chloroquine (CQ) diluted in PBS was added to result in a final concentration of 10 μM (24 h of exposure) or 50 μM (4 h of exposure). Some experiments included incubations with inductors (50 μM Rapamycin, 100 μM Resveratrol) of the autophagic flux, in presence or absence of CQ. All experiments were carried out in 10% FBS, as fasting constitutes an additional stimulus for autophagy.

For reoxygenation, hypoxic medium was replaced for standard medium and cells were incubated for 1 h at 37°C with 5% CO_2_.

### MTT Assay

To determine cell viability, the colorimetric MTT assay was performed. Briefly, 2.000 cells per well were seed in a 96-well plate and incubated under different experimental conditions (normoxia, hypoxia, CoCl_2_, with or without Rapamycin or Resveratrol) for 24 h. Then, 10 μl of the yellow tetrazolium salt 3-(4, 5-dimethylthiazol-2-yl)-2, 5-diphenyltetrazolium bromide (MTT) (5 mg/ml) was added to culture medium. Cells were incubated with the MTT reagent for additional 3 h at 37°C. After that, cell medium was carefully removed and 100 μl of DMSO was added in order to solubilize the crystal violet. Finally, the optical density values were measured at a wavelength of 570 nm using a SpectraMax M5 plate reader (Molecular Devices, United States).

### Western Blot Assay

Following treatment, cells were lysed with a solution of Triton X-100 1% in PBS, 1 mM PMSF and protease inhibitor cocktail (Sigma Aldrich, St. Louis, MO). Lysates were cleared of insoluble material by centrifugation at 15,000 g for 10 min at 4°C. Protein concentration of MIO-M1 cell extracts were measured by a Bicinchoninic Acid Protein Assay Kit (Pierce BCA, Thermo Scientific, United States) according to the manufacturer’s protocol and 20 μg of proteins were electrophoresed in 15% SDS-PAGE. After electrophoresis, proteins were transferred to nitrocellulose membranes (Amersham Hybond ECL; GE Healthcare Bio-Sciences AB, Uppsala, Sweden). To prevent nonspecific binding, membranes were blocked with 5% milk in TBS containing 0.1% Tween-20 (TBST) during 1 h at room temperature (RT). Then, blots were incubated with primary antibodies diluted in 1% BSA in TBST overnight at 4°C. The following antibodies were used: rabbit polyclonal anti-LC3 (1/1000; L7543, Sigma Aldrich), mouse monoclonal anti p62 (1/1000; ab56416, Abcam), rabbit polyclonal anti-GFAP (1/1000; Dako, Carpinteria, CA), mouse monoclonal anti-Vimentin (1/1000; M7020, Dako) and mouse monoclonal anti-β-actin (1/2000; ab8226, Abcam). Blots were incubated with IRDye 800 CW donkey anti-rabbit Ig, or IRDye 800 CW donkey anti-mouse IgG antibodies (1/15000 in TBS with 5% BSA) for 1 h, protected from light. After washing with TBST, membranes were visualized and quantified using the Odyssey Infrared Imaging System (LI-COR, Inc., Lincoln, NE, United States).

### Immunofluorescence Assays and Confocal Microscopy

Briefly, cells were grown at 30% confluence on coverslips. For staining with the lysosome marker LysoTracker Red (Thermo Fisher Scientific) 1 μl/ml of the reagent was added to the cell medium in the final hour of the incubation time. After stimuli, coverslips were washed twice with precooled PBS and fixed in PBS containing 4% PFA/4% sucrose for 15 min at RT. Free aldehyde groups were blocked with 50 mM NH_4_Cl in PBS during 20 min. The cells were then incubated in a blocking buffer (PBS containing 0.05% saponin/0.2% BSA) for 30 min. Antibodies were prepared in blocking buffer. Cells were then incubated overnight at 4°C with the following primary antibodies: rabbit polyclonal anti-LC3 (1/100; L7543, Sigma Aldrich), mouse monoclonal anti p62 (1/100; ab56416, Abcam). Then, cells were washed three times with PBS plus 1% BSA, and exposed to secondary antibodies diluted 1:800 for 45 min at RT. After a thorough rinse with PBS plus 1% BSA, cell nuclei were also counterstained with Hoechst 33258 (1:1000; Molecular Probes) for 7 min. Cells were washed with PBS and mounted on glass slides with Mowiol 4–88 (Calbiochem; Merck KGaA, Darmstadt, Germany). Fluorescent images were obtained with a confocal laser-scanning biological microscope (Olympus FluoView FV1200; Olympus Corp., New York, NY, United States). Finally, images were processed with ImageJ FIJI software (National Institutes of Health, Bethesda, MD, United States).

### qRT-PCR

Total RNA was extracted from cultured cells using Trizol Reagent (Invitrogen) (Ridano et al., 2012). Briefly, 1 μg of total RNA was reverse-transcribed in a total volume of 20 μl using random primers (Invitrogen, Buenos Aires, Argentina) and 50 U of M-MLV reverse transcriptase (Promega Corp.). Then, cDNA was mixed with 1x SYBR Green PCR Master Mix (Applied Biosystems) and forward and reverse primers: Beclin-1 forward: CCATGCAGGTGAGCTTCGT/Beclin-1 reverse: GAA​TCT​GCG​AGA​GAC​ACC​ATC; ATG5 forward: AAA​GAT​GTG​CTT​CGA​GAT​GTG​T/ATG5 reverse: CAC​TTT​GTC​AGT​TAC​CAA​CGT​CA. VEGF-A forward:CCGCAGACGTGTAAATGTTCCT/VEGF-A reverse: CGG​CTT​GTC​ACA​TCT​GCA​AGT​A; PEDF forward: GCTGAGTTACGAAGGCGAAGT/PEDF reverse: TTG​ATG​GGT​TTG​CCT​GTG​AT. qPCR were carried out on an Applied Biosystems 7500 Real-Time PCR System with Sequence Detection Software v1.4. The cycling conditions included a hot start at 95°C for 10 min, followed by 40 cycles at 95°C for 15 s and 60°C for 1 min. Specificity was verified by melting curve analysis. Results were normalized to β-actin (Forward: AAATCTGGCACCACACCTTC/Reverse: GGG​GTG​TGA​AGG​TCT​CAA​A). Relative gene expression was calculated according to the ^2−ΔΔCt^ method. Each sample was analyzed in triplicate. No amplification was observed in PCRs using as template water (data not shown).

### Tube Formation Assay

The assay was performed according to Arnaoutova y Kleinman, (Arnaoutova and Kleinman, 2010). Briefly, BAEC (≈1.5 × 10^4^ cells) were placed on a 96 well half area plate previously coated with 30 uL of Matrigel. The plates were incubated for 18 h (37°C, 5% CO_2_) in the presence of normoxic or hypoxic MIO-M1 supernatants containing vehicle or autophagy inductors. Sodium suramine 30 uM and Axitinib 10 uM were used as positive control of inhibition. Control samples with and without their respective vehicles were simultaneously run. The images were obtained with an Olympus CKX41 inverted microscope and analyzed with the software ImageJ. The tubular structures were quantified and the percentages of inhibition (I%) were calculated I% = [1−(Total tube length treatment/Total tube length control)] x 100.

### Statistical Analysis

Statistical analysis was performed using the GraphPad Prism 7.0 software. A p-value < 0.05 was considered statistically significant. Parametric or nonparametric tests were used according to variance homogeneity evaluated by F or Barlett’s tests. Two-tailed unpaired t or Mann Whitney tests were used in analysis of two groups. One-way analysis of variance (ANOVA) followed by Dunnett’s multiple comparison post-test or Kruskal-Wallis followed by Dunn´s multiple comparison post-test were used to determine statistical significance among more than two different groups. Mean ± standard error (SEM) are shown in graphs analysed with parametric tests and median with interquartile range are shown when data were analysed with nonparametric test.

## Results

### Autophagic Flux is Increased in MIO-M1 Cells Exposed to 4 h of Hypoxia

Previous studies conducted in MGCs exposed to hyperglycemia showed an altered autophagic flux given that autophagosomes could not reach the degradation step (Lopes de Faria et al., 2016). However, in a hypoxic environment rat MGCs displayed an increase in LC3B II protein levels (Fung et al., 2016). Thereby, our first aim was to evaluate changes in the autophagic flux in human MGCs (MIO-M1), under hypoxia. Two experimental hypoxic models were used: the gas culture chamber (cells exposed to 1% O_2_) and a chemical model (cells exposed to CoCl_2_). Our experimental design included controls with and without Chloroquine (CQ) in order to assess the accumulation of the autophagosomes.

**FIGURE 1:**
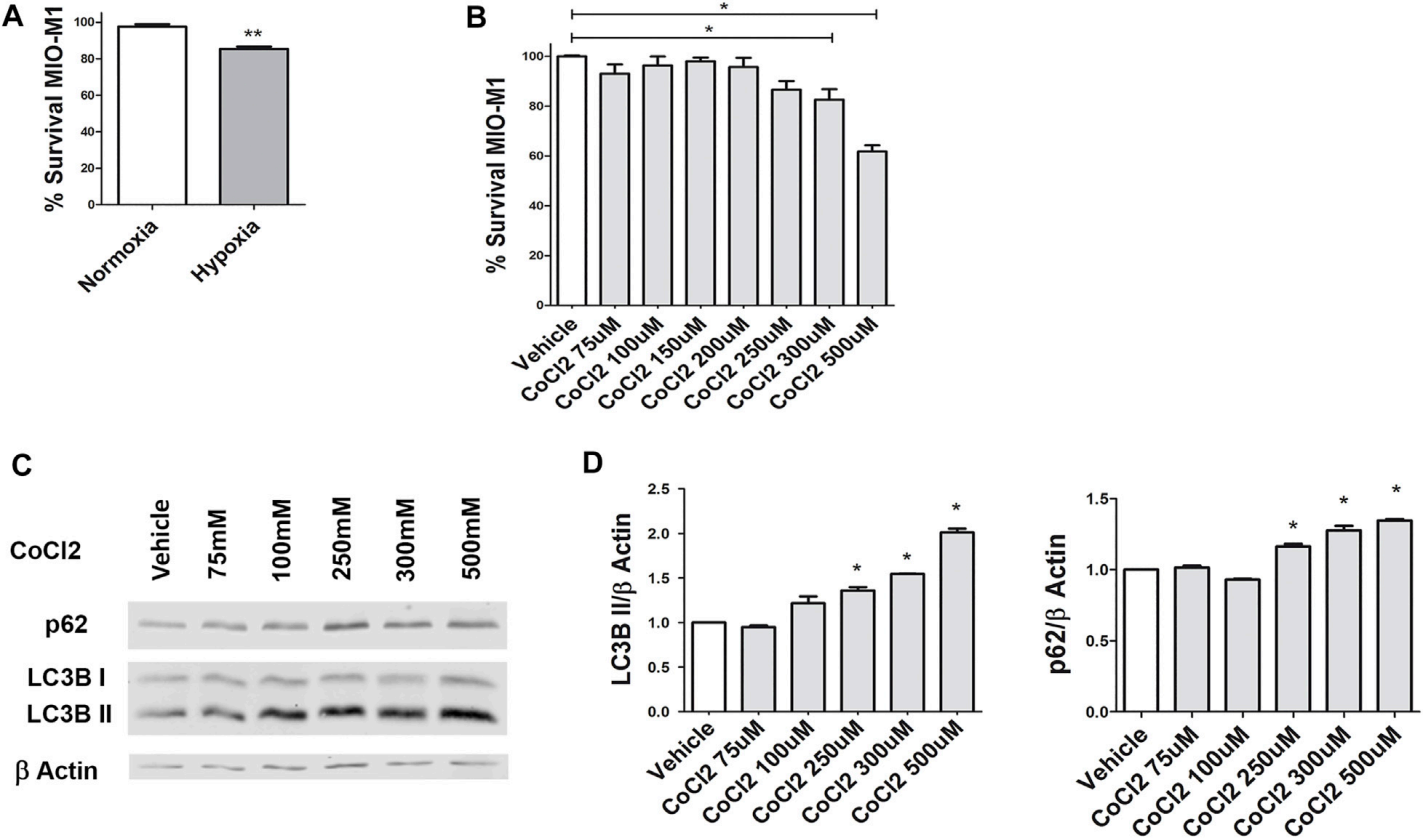
Effect of hypoxia (gas or CoCl_2_) on human MIO-M1 viability by MTT assay. **(A)** Viability of MIO-M1 cells exposed to 24 h hypoxia (1% O_2_) or normoxia. **(B)** Viability of MIO-M1 cells exposed to increasing concentrations of CoCl_2_ during 24 h. The cell viability was evaluated by MTT assay and measured by spectrophotometry. **(C)** Representative Western blot of autophagy markers, LC3B II and p62, from cell lysates of MIO-M1 incubated with increasing concentrations of CoCl_2_ during 4 h β-actin is shown as a loading control. **(D)** Protein levels of LC3B II and p62 were quantified by densitometry and normalized by β actin. Data is presented as mean ± SEM. *p < 0.05, **p < 0.01. Graph shows results of three independent experiments.

Although MGCs are very perceptive to environmental changes, they are considered the most resistant retinal cells to external injuries (Coughlin et al., 2017). In order to examine MIO-M1 survival under both hypoxic models, we carried out MTT assays at 24 h (Figure 1). MIO-M1 viability was reduced by 15% when exposed to 1% O_2_ compared to normoxia (*p* = 0.001) (Figure 1A). For chemical hypoxia, we tested increasing concentrations of CoCl_2_. Viability of MIO-M1 cells exposed to CoCl_2_ up to 250 μM did not change significantly respect to the control (*p* = 0.1358) (Figure 1B). Of note, no significant differences were observed in the MIO-M1 viability for shorter exposure periods (4 and 8 h, data not shown). Then, we studied changes in the expression of autophagic proteins in MIO-M1 cells exposed to CoCl_2_ for 4 h by Western blot (Figure 1C). LC3B II (a structural protein of the autophagosomes) and p62 (an adaptor protein that is degraded with the autophagosome content) were evaluated. The quantitative analyses of LC3B II and p62 bands evidenced a significant increase in the autophagic protein levels since a concentration of 250 μM respect to vehicle (*p* = 0,027 for LC3B II and *p* = 0,031 for p62). Therefore, we selected 250 μM CoCl_2_ as a concentration for *in vitro* studies as it represents the minimum concentration that achieves two relevant requisites: it is not detrimental for cell viability and it is able to induce the autophagic flux.

**FIGURE 2:**
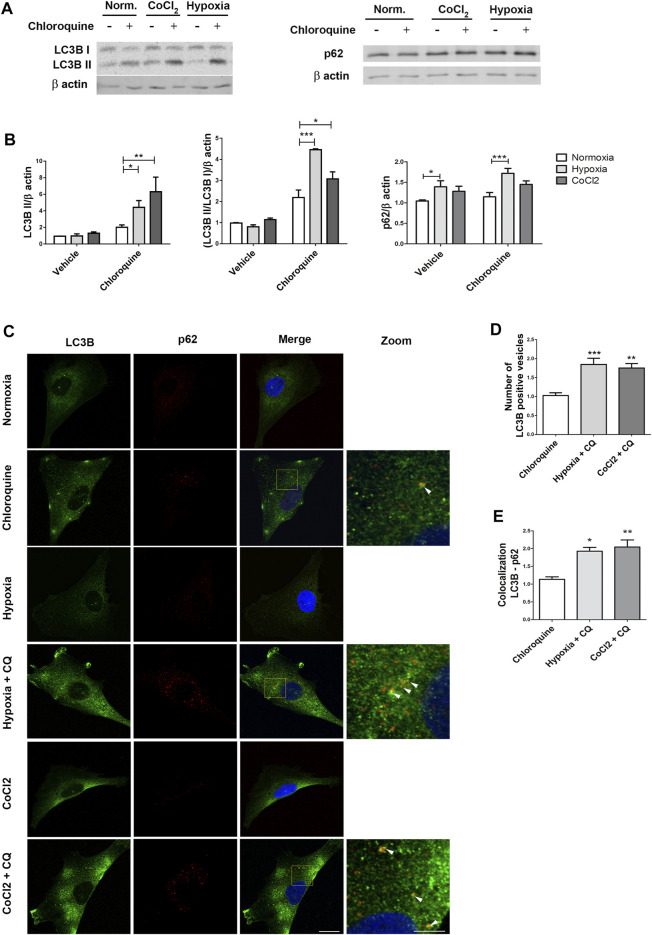
Hypoxia increases LC3B II and p62 protein levels in MIO-M1. **(A)** Representative Western blot of autophagy markers, LC3B II and p62, from cellular lysates of MIO-M1 incubated in hypoxic conditions: gas (1% O_2_) or chemical hypoxia (250 mM CoCl_2_) during 4 h. **(B)** Protein levels of LC3B II and p62 were quantified by densitometry and normalized to β actin. Graph shows results of three independent experiments. **(C)** Representative immunofluorescence analysis of LC3B (green) and p62 (red) in MIO-M1 cells incubated in hypoxic conditions: gas (1% O_2_) or CoCl_2_ (250 mM) during 4 h. Cell nuclei were counterstained with Hoechst 33258 (blue). Images were taken with oil 60X objective in the best confocal resolution condition. **(D)** Quantification of LC3B puncta per cell with ImageJ analyze particles software. Values were normalized to normoxia + chloroquine. **(E)** Quantification of LC3B/p62 colocalization with ImageJ JACoP software. Pearson values were compared statistically. Values were normalized to normoxia + chloroquine. Statistical t test was performed. White arrows in the images show mayor colocalization areas. Data is presented as mean ± SEM. *p < 0.05, **p < 0.01, ***p < 0.001. Graph shows results of three independent experiments.

Autophagy is a catabolic process rapidly activated in response to environmental stimuli that enables the cell adaptation to the new living conditions (Mizushima and Komatsu, 2011). Hence, we decided to evaluate if hypoxia modified the autophagic flux in human MGCs exposed to short periods. For this purpose, the MIO-M1 cells were exposed to hypoxia or normoxia during 4 h, in presence of CQ or vehicle in the above indicated conditions. Simultaneously, a comparative study was performed by incubating MIO-M1 cells with CoCl_2_ 250 μM, including controls with and without CQ (Figure 2). By Western blot assay we observed no significant changes in LC3B II and p62 protein expression levels in gas hypoxia or CoCl_2_ respect to normoxia in absence of CQ. However, in samples treated with CQ a significant increase in LC3B II protein levels was observed under both hypoxic treatments (*p* = 0.0447 for 1% O_2_ and *p* = 0.0021 for CoCl_2_) respect to normoxia.

Under 1% O_2_, MIO-M1 cells incubated with CQ showed a significant increase in p62 protein levels (*p* = 0.0006) (Figures 2A,B) respect to normoxia-CQ. Although we observed a non-significant increase in the expression of p62 in presence of CoCl_2_ 250 μM (*p* = 0.3959), incubation with CQ showed a significant accumulation of LC3B II in MIO-M1 cells respect to vehicle, suggesting that the autophagic vesicles follow the degradation pathway properly.

Correlatively, immunofluorescence assays with classic markers of autophagy showed an increased number of LC3B puncta per cell in MIO-M1 exposed to gas hypoxia (*p* < 0.001) and CoCl_2_ (*p* = 0.005), respect to normoxia (Figures 2C,D). In line with these results, a higher colocalization of LC3B and p62 was determined in both hypoxic conditions respect to normoxia (*p* = 0.018 for 1% O_2_ and *p* = 0.0069 for CoCl_2_) (Figure 2E).

### Autophagic Flux Returns to Normoxic Levels After 24 h of Hypoxia in MIO-M1 Cells

Cell response to an injurious stimulus can vary depending on its duration. Therefore, we decided to evaluate MGCs behaviour under prolonged exposure to hypoxia (24 h) by analysing changes in their autophagic flux. Experimental conditions were similar to those in shorter hypoxic stimuli. By Western blot assays, similar protein expression levels of LC3B II and p62 were observed in MIO-M1 cells under gas hypoxia and normoxia when incubated with CQ (*p* > 0.9999 for LC3BII and *p* = 0.2821 for p62) (Supplementary Figures S1A,B). Then, we quantified mRNA levels of Beclin-1 and ATG5. Results showed a not statistically significant decrease in Beclin-1 and ATG-5 transcript levels in MIO-M1 cells under 1% O_2_ (*p* = 0.1248 for Beclin-1 and *p* = 0.0753 for ATG5) (Supplementary Figure S1C). In contrast, an increase in autophagy protein levels was observed in cells incubated with CoCl_2_ for 24 h in presence of CQ (*p* = 0.0011 for LC3BII and *p* < 0.0001 for p62) (Supplementary Figures S1A,B). In spite of this result, mRNA levels of Beclin-1 and ATG5 were not modified (*p* = 0.7368 for Beclin-1 and *p* = 0.3638 for ATG5), suggesting a slight activation of the autophagic flux (Supplementary Figure S1C). Confocal images of MIO-M1 cells exposed to hypoxia (1% O_2_) and normoxia revealed similar number of LC3B puncta per cell (*p* = 0.3081) and colocalization of LC3B and p62 (*p* = 0.2975) (Supplementary Figures S1D–F). On the other hand, CoCl_2_ induced an increase in the number of vesicles decorated with LC3B (*p* = 0.0373) and a slight rise in the colocalization of LC3B and p62 respect to control (*p* = 0.0024) (Supplementary Figures S2A–C). These results demonstrate a higher number of autophagosomes when MIO-M1 cells were exposed to CoCl_2_ for 24 h respect to control. The late stage of the autophagy pathway involves the degradation of the autophagosome content and its inner membrane by fusion with lysosomes. In order to evidence the fusion of autophagosomes with lysosomes, immunofluorescence assays were performed with LC3B and LysoTracker, an acidic compartment and late endosomes marker. The colocalization analysis evidenced no difference in the overlap of the proteins when MIO-M1 cells were incubated with vehicle or CoCl_2_ during 24 h (*p* = 0.9738) (Supplementary Figures S2D,E). Moreover, we also evaluated the lysosomal membrane integrity by immunostaining with Galectin-1 (Gal-1). In our experiments, Gal-1 staining showed a homogeneous cytoplasmic distribution in MIO-M1 cells in both hypoxic models, suggesting that lysosomal structure was preserved after 24 h of exposure (Supplementary Figure S2F).

### Autophagic Flux is Increased in MIO-M1 Cells After Reoxigenation

**FIGURE 3:**
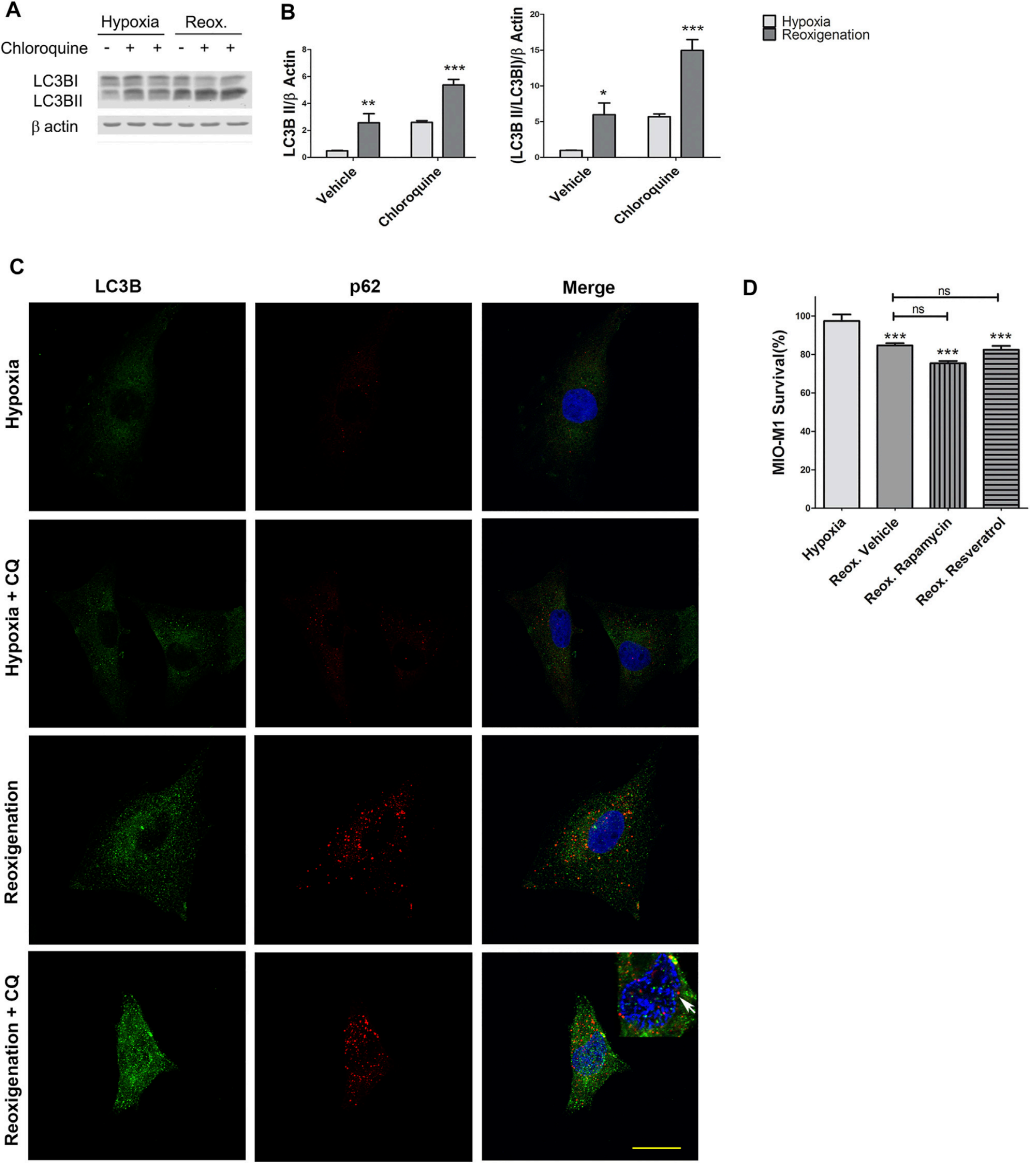
Reoxygenation increases LC3B II and p62 protein levels in MIO-M1. **(A)** Representative Western blot of autophagy marker LC3B II from cellular lysates of MIO-M1 incubated in hypoxic conditions (1% O_2_) during 4 h and later returned to normoxia for 1 h (reoxygenation). **(B)** Protein levels of LC3B II were quantified by densitometry and normalized to β actin. **(C)** Representative immunofluorescence analysis of LC3B (green) and p62 (red) in MIO-M1 incubated in hypoxic conditions (1% O_2_) during 4 and 1 h of reoxygenation. Cell nuclei were counterstained with Hoechst 33258 (blue). Images were taken with oil 60X objective in the best confocal resolution condition. **(D)** Evaluation of the viability of MIO-M1 cells in hypoxic conditions or hypoxia-reoxygenation and incubated with vehicle, Rapamycin or Resveratrol. Data is presented as mean ± SEM. *p < 0.05, **p < 0.01, ***p < 0.001. Graph shows results of three independent experiments.

Clinical studies provided evidence about the variation of oxygen levels in neuronal tissues in patients with PDR (Daulatzai, 2017). Hence, we next evaluated the autophagic flux in MIO-M1 cells exposed to 4 h hypoxia followed by 1 h of reoxygenation. Quantitative analysis of Western blot assays revealed a significant increase in LC3B II (*p* < 0.001) in reoxygenated MIO-M1 cells compared to the hypoxic ones, in conditions with CQ (Figures 3A,B). In line with this results an increase in vesicles decorated by LC3B were observed in reoxygenated MIO-M1 cells in presence of CQ (Figure 3C), suggesting an increase in the number of autophagosomes when returned to normoxia after the hypoxic period. To determine the role of autophagy in reoxygenation, we assessed MIO-M1 survival by MTT assay (Figure 3D). The cell viability of the reoxygenated MIO-M1 cells was significantly lower than in hypoxic condition (*p* = 0.0009). In addition, inductors of the autophagic flux were unable to restore MIO-M1 survival (*p* = 0.305 for Reoxygenation + Rapamycin; *p* = 0.9999 for Reoxygenation + Resveratrol). Notably, the blockade of the flux with CQ induced cytoplasmatic retraction and nuclear condensation in MIO-M1 cells under reoxygenation (Figure 3C, white arrow).

### MIO-M1 Cells Gliosis is Decreased by Autophagy Inductors

In response to an injury or stressor, MGCs trigger protective mechanisms in order to preserve retinal structure and functionality, a process called reactive gliosis (Subirada et al., 2018). We have previously demonstrated in a mouse model of Oxygen-induced retinopathy (OIR) that MGCs become reactive after the hyperoxic phase and modified the expression of stress and detoxifying proteins from postnatal days 12 to 26 in OIR mice (Ridano et al., 2017). Then, our next aim was to analyse changes in the expression levels of GFAP after hypoxic exposure. This set of experiments was performed with gas hypoxia, at the time where a main response to hypoxia was detected (4 h).

**FIGURE 4:**
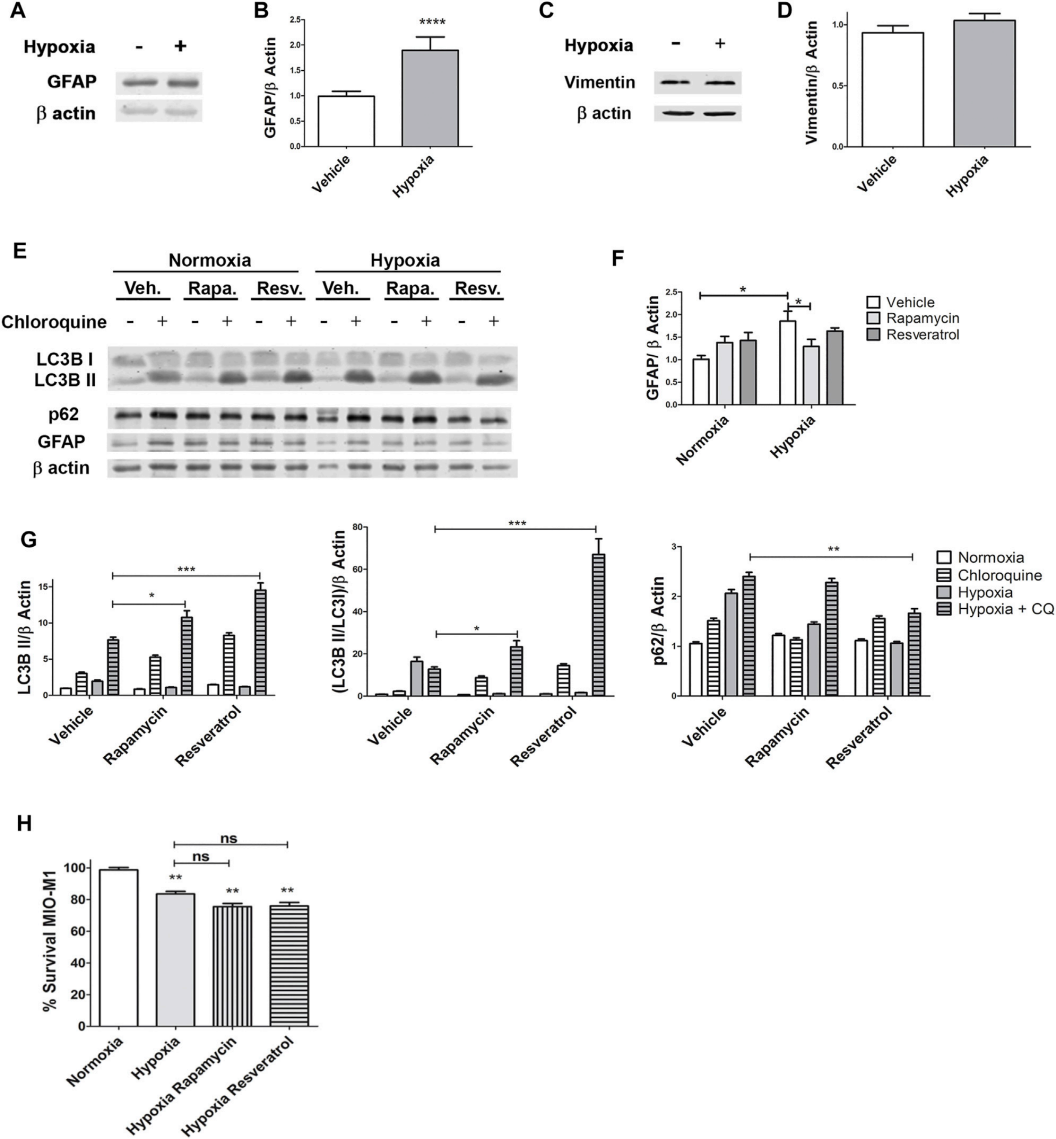
Rapamycin decreases GFAP protein expression in MIO-M1. **(A)** Representative Western blot of GFAP from cellular lysates of MIO-M1 incubated in hypoxic conditions (1% O_2_) during 4 h. **(B)** Protein levels of GFAP were quantified by densitometry and normalized to β actin. **(C)** Representative Western blot of Vimentin from cellular lysates of MIO-M1 incubated in hypoxic conditions (1% O_2_) during 4 h. **(D)** Protein levels of vimentin were quantified by densitometry and normalized to β actin. **(E)** Representative Western blot of GFAP and autophagy markers, LC3B II and p62, from cellular lysates of MIO-M1 incubated in normoxia or hypoxia (1% O_2_) during 4 h (with or without CQ) and in presence of vehicle, Rapamycin or Resveratrol. **(F)** Protein levels of GFAP were quantified by densitometry and normalized to β actin. **(G)** Protein levels of LC3B II and p62 were quantified by densitometry and normalized to β actin. **(H)** Evaluation of the viability of MIO-M1 cells in normoxia or hypoxia during 24 h and incubated with vehicle, Rapamycin or Resveratrol. Graph shows results of three independent experiments. Data is presented as mean ± SEM. *p < 0.05, **p < 0.01, ***p < 0.001, ****p < 0.0001.

After 4 h of hypoxia, MIO-M1 cells showed an increase in protein levels of GFAP (*p* < 0.0001) in accordance with the activation of glial cells (Figures 4A,B), although no differences in Vimentin expression was detected (*p* = 0.1012) by Western blot assays (Figures 4C,D).

We have previously demonstrated in the OIR mouse model, a decrease in the GFAP protein levels after a single intraocular injection of Rapamycin at postnatal day 26 OIR (Subirada et al., 2019). Therefore, we aimed to determine if inductors of the autophagic flux prevented the increase of GFAP expression under hypoxic conditions. For this purpose, MIO-M1 cells were incubated in normoxia or hypoxia (1% O_2_) and in presence of Rapamycin or Resveratrol for 4 h. Western blot assays showed a substantial decrease in GFAP protein expression levels by Rapamycin in MIO-M1 cells exposed to 1% O_2_ (*p* = 0.0438). A trend to diminish GFAP protein levels was observed in hypoxia/Resveratrol, however, this decrease was not statistically significant (*p* = 0.6558) (Figures 4E,F). We confirmed the induction of the autophagic flux in this experiment by analysis of LC3B II and p62 protein levels (Figures 4E,G). Lastly, we evaluated if autophagy inductors could prevent cell death after 24 h of hypoxia, the only experimental time point where decreased viability was observed (Figure 1A). The MTT assay showed that MIO-M1 cells incubated with either of the inductors exhibited similar cell survival respect to hypoxia vehicle (*p* = 0.0834 for hypoxia + Rapamycin; *p* = 0.1337 for hypoxia + Resveratrol) (Figure 4H).

### MIO-M1 Cells Modulate Angiogenesis in Presence of Autophagy Inductors

**FIGURE 5:**
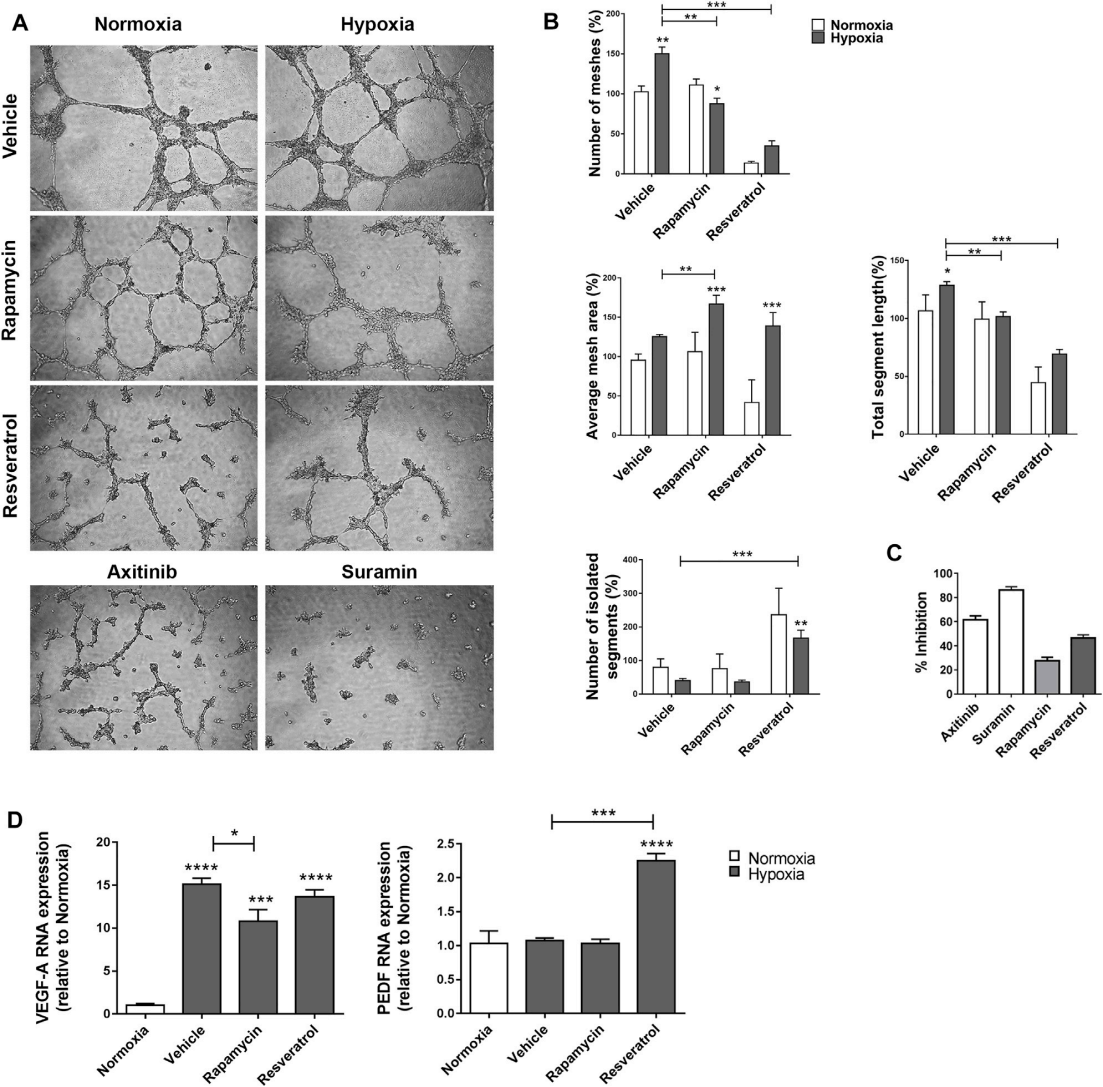
Supernatants of MIO-M1 incubated with Rapamycin or Resveratrol inhibit BAEC tubulogenesis. **(A)** Contrast phase images of BAEC incubated with MIO-M1 supernatants in normoxic or hypoxic conditions during 8 h and in presence of vehicle, Rapamycin or Resveratrol. Axitinib and Suramin were used as positive control of tubulogenesis inhibition. **(B)** Quantitative analysis of the number and average area of meshes, total segment length and the number of isolated segments of BAEC performed with Image J Fiji Angiogenesis Analyser Software. **(C)** Percentage of tubulogenesis inhibition mediated by Suramin, Axitinib, Rapamycin or Resveatrol, respect to control. **(D)** VEGF and PEDF mRNA levels were quantified by qRT-PCR in MIO-M1 cells in normoxia or hypoxia and in presence of vehicle, Rapamycin or Resveratrol. Results were normalized to β-actin and expressed according to the ^2−ΔΔCt^ method using as calibrator the mRNA level obtained from normoxia. Graph shows results of three independent experiments. Data is presented as mean ± SEM. *p < 0.05, **p < 0.01, ***p < 0.001, ****p < 0.0001.

It is known that MGCs maintain the vascular structure through a balance between synthesis and secretion of pro- and anti-angiogenic factors. Thus, our next goal was to evaluate MGCs ability to induce tubulogenesis of endothelial cells (ECs) under hypoxia and how the inductors of the autophagy modified this response. For this purpose, we incubated MIO-M1 cells under normoxia or hypoxia (1% O_2_) with an inductor of the autophagic flux or vehicle, and obtained their supernatants. Then, tubulogenesis assays were performed on Matrigel by incubating ECs with the supernatants of MIO-M1 cells during 24 h (Figure 5A). Suramin and Axitinib were used as negative controls of tubulogenesis. Quantitative analysis of the tubular network formed by ECs showed that hypoxic MIO-M1 supernatants increased the formation of vascular tubules respect to normoxic supernatants. We observed an increase in the mesh number (polygonal vascular structures) and the total segment length of vessel tubules in ECs incubated with hypoxic supernatants (Figures 5A,B). Then we compared ECs tubulogenesis incubated with supernatants containing vehicle respect to those containing an autophagy inductor. MIO-M1 supernatants exposed to hypoxia and in presence of Rapamycin decreased the mesh number, increased the average mesh area and decreased the total segment length respect to hypoxic + vehicle supernatant. Those changes were not observed between vehicle and Rapamycin in normoxic supernatants. On the other hand, Resveratrol modified all the quantified parameters, showing an evident decrease in the tubule formation respect to vehicle. Resveratrol had a similar effect when ECs were incubated with both normoxic and hypoxic MIO-M1 supernatants. In line with these results, both treatments in hypoxic supernatants showed a significant increase in the percentage of tubulogenesis inhibition respect to hypoxic-vehicle supernatant (Figure 5C). Finally, we examined if autophagy inductors had a direct effect over ECs. For that purpose, we incubated ECs with Rapamycin and Resveratrol for 24 h. We observed no variations in the formation of polygonal structures when ECs were incubated with either autophagy inductor respect to vehicle (Supplementary Figure S3).

It has been demonstrated that under hypoxic conditions MGCs are one of the cells responsible for the increase of VEGF retinal levels, the main pro-angiogenic factor involved in angiogenesis (Wang et al., 2010). To restrict the vascular growth, MGCs secrete PEDF, which counterbalance pro-angiogenic factors (Yang et al., 2012). In order to determine if trophic factors levels were modified by the autophagy inductors, we further quantified the transcript levels of VEGF and PEDF in hypoxic MIO-M1 cells (Figure 5D). Quantitative RT-PCR analysis showed an increase in VEGF mRNA levels in hypoxia respect to normoxia. Moreover, Rapamycin induced a significant decrease of VEGF mRNA respect to hypoxia vehicle (*p* = 0.0334). VEGF transcript was not modified in MIO-M1 incubated in hypoxia + Resveratrol. By contrast, PEDF mRNA was only increased in hypoxic MIO-M1 cells treated with Resveratrol (*p* = 0.0003). In summary, the results indicate that Rapamycin and Resveratrol prevents tubule formation by modulating different proteins involved in angiogenesis.

## Discussion

MGCs provide neuronal support and communicate with other cells in order to maintain retinal homeostasis. Their roles in health and disease are indispensable for neuronal survival, however, they also undergo changes and eventually die after an injury. Autophagy is a catabolic pathway that can be up- or down-regulated in ischemic retinopathies. The increase in the autophagic flux has been linked to cell survival as it removes ROS or damaged organelles and proteins (Kroemer et al., 2010). However, under specific circumstances, excessive activation can also lead to cell death. With a proper pharmacological therapy, autophagy modulators have prevented neurodegeneration in several animal models (Lin and Xu, 2019). Autophagy inductors are a group of compounds that are able to increase autophagy flux. Their effects are exerted by the regulation of different signaling pathways that modulate autophagic flux but also by inducing other cellular events (Li et al., 2014; Zhou et al., 2018).

In a previous study we showed that induction and inhibition of the autophagic flux had a direct impact on vascular and glial events in the experimental OIR mouse model (Subirada et al., 2019). Here, we evaluated the ability of two autophagy inductors to modulate MCG functions under hypoxia.

Initially, we evaluated variations in the autophagic flux of human MGCs under two hypoxic models (gas and chemical during 4 h). In both models, we observed augmented LC3B II protein levels respect to normoxia in presence of CQ, suggesting an increase in the autophagic flux. The accumulation of autophagosomes in presence of CQ respect to their corresponding vehicle controls demonstrate that the autophagic vesicles can reach the final stage of the degradation pathway. Quantitatively, the increase in LC3B II protein levels in MIO-M1 cells was higher under CoCl_2_ than under gas hypoxia, indicating that the nature of the stimulus influences the intensity of the response. The increase in the number of LC3B vesicles and colocalization of LC3B and p62 reinforced these results as a major number of autophagosomes was observed in MIO-M1 cytoplasm under chemical and gas hypoxia. Similar results were obtained when incubating MIO-M1 cells under gas hypoxia. Although this work did not focus in the signalling pathways that activate autophagy under hypoxia, experiments performed with CoCl_2_ constitute a reflex of the activation of the flux via HIF-1α (Yuan et al., 2003). Respect to gas hypoxia, it is possible that other signalling pathways may also be activated (Li et al., 2015).

Autophagy is a catabolic mechanism by which altered proteins, organelles and others are recycled to amino acids in different stressful conditions, contributing to the reestablishment of the homeostasis (Klionsky et al., 2021). Thereby, it would be predictable that the autophagic flux have a relevant role during initial stages of the stimulus, until the cell reach a stable metabolic state. Prolonged exposition frequently show a different autophagy flux (Zhang et al., 2020). By Western blot and immunofluorescence assays we demonstrated that autophagic flux returned to normoxic levels when incubated in 1% O_2_ during 24 h. This result indicates that the activation of the flux is transient and takes place immediately after the exposition to hypoxic environment. Conversely, LC3B II and p62 protein levels remained elevated after a 24 h stimulus with CoCl_2_. The increase in the ratio of LC3B II to LC3B I evidenced that the autophagic flux is still activated. At this final time point, a similar colocalization between LC3B and Lysotracker was observed in normoxic and hypoxic conditions, demonstrating that the fusion of autophagosomes and lysosomes is not altered by CoCl_2_. On the other hand, a Gal-1 dotted pattern could be indicative of permeability and subsequent lysosomal dysfunction. No changes in Gal-1 staining pattern were detected under both hypoxic conditions respect to normoxia. All conditions exhibited a homogeneous cytoplasmic distribution of Gal-1, indicating that lysosomes are not permeable.

In this work, we also explored the variation in the autophagic flux after reoxygenation. Insulin resistance and metabolic derangement have been linked to sleep apnea and other sleeping disorders observed in diabetics, which can further induce nocturnal events of hypoxia and reoxygenation in the retina (Shiba et al., 2010; Thomas et al., 2017). As assessed by Western blot and confocal images, there is an increase in autophagosomes in MIO-M1 cells when reoxygenated respect to hypoxia. Moreover, the accumulation of LC3B II in hypoxia and reoxygenation with CQ respect to vehicle proved that there is no alteration in the degradation pathway in both experimental conditions. The restoration of oxygen levels to normoxia reduced the survival of MIO-M1 cells, probably due to the fast activation of the respiratory chain in presence of O_2_ and subsequent production of reactive oxygen species. In this sense, the activation of autophagy could represent a protective response to oxidative stress damage. Unfortunately, autophagy results insufficient to preserve the cell survival, as observed by the addition of Rapamycin and Resveratrol. This poorly studied effect of oxygen levels variation at night would partly explain the neurodegeneration observed in the retina of PDR diabetic patients even after anti-angiogenic treatment.

The gliotic response triggered after an injury provides protection to all retinal cells. Key features in the gliotic response are the biochemical and morphological changes experienced by MGCs, which include the up-regulation of intermediate filaments and detoxification enzymes (Graca et al., 2018). In our cellular system, incubations at low oxygen levels for 4 h increased GFAP protein expression, a glial stress marker. Other filamentous protein, Vimentin, showed less sensibility to the hypoxic exposure, as its protein levels remained constant respect to normoxia. It has been shown that Rapamycin treatment decreased astroglial reactivity in several neurological pathologies (Siman et al., 2015; Liu et al., 2021). In our hypoxic MIO-M1 culture, we observed decreased GFAP expression after Rapamycin treatment, whereas Resveratrol showed a non-significant reduction in this protein levels. We consider that the underlying mechanisms to this event could be different between both inductors, and even independent of the modulation of the autophagic flux. Overall, the ability of Rapamycin to prevent gliosis constitute a potential pharmacological strategy to cope with persistent gliosis, a relevant aspect of chronic ischemic retinopathies that is not improved with anti-VEGF treatment (Ridano et al., 2017). Unfortunately, at the concentrations evaluated, Rapamycin and Resveratrol were not able to mitigate cell death. Because the similar cell viability was detected in presence and absence of the inductors, we can conclude that autophagy is not the cell death mechanism involved in hypoxic cell death, but its activation is not sufficient to prevent MCGs death.

Finally, we examined another MGC function under hypoxia: its participation in vascular proliferation. A reduced formation of tubular structures was observed when ECs were incubated with MIO-M1 supernatants exposed to hypoxia and in presence of Rapamycin. The observation that normoxic supernatants with Rapamycin were not able to decrease tubulogenesis, demonstrates that Rapamycin anti-proliferative effect is exclusively exerted under hypoxia. The balance towards the anti-proliferative effect can be shifted by increasing the synthesis of anti-angiogenic proteins (as PEDF) or by decreasing the synthesis of the pro-angiogenic proteins (mainly VEGF) (Yafai et al., 2007). Accordingly, Rapamycin reduced VEGF mRNA as it was previously described (Liu et al., 2015; Kida et al., 2021). Instead, Resveratrol strongly inhibited ECs tubulogenesis in both hypoxic and normoxic MIO-M1 supernatants. In this regard, some studies have previously described the ability of Resveratrol to increase PEDF synthesis (Hua et al., 2011; Ishida et al., 2017). As expected, PEDF levels were increased in hypoxic conditions after Resveratrol treatment, suggesting an anti-angiogenic cell response. At the concentrations employed in this work, we did not observe a variation in the formation of tubular structures when the inductors were incubated directly with ECs. This result demonstrates that the antiangiogenic effects observed are mediated by MIO-M1 response.

In this research, we evaluated MGCs autophagy flux in two experimental hypoxic models, confirming the activation of an early autophagic response. At the same time, MGCs established a gliotic response and participated in vaso-proliferative events. Inductors of the autophagy flux partially reverted these responses, considering Rapamycin as the compound that more beneficial effects produced (Supplementary Table S1). Its ability to decrease persistent gliosis and reduce VEGF synthesis shows that Rapamycin modulates vascular and non-vascular alterations through direct effect on MGCs, two relevant aspects of ischemic retinopathies. Our results provide a general overview of the multiple benefits of MGCs modulation with autophagy inductors.

## Data Availability

The original contributions presented in the study are included in the article/Supplementary Material, further inquiries can be directed to the corresponding author.

## Nomenclature

ATG-5: Autophagy related protein 5
BAECs: Bovine Aortic Endothelial Cells
**CoCl**_**2**_: Cobalt chloride
CQ: Chloroquine
ECs: endothelial cells
Gal-1: Galectin-1
GFAP: Glial fibrillary acid protein
HIF-1: Hypoxia inducible factor 1
LC3B: Microtubule-associated proteins 1A/1B light chain 3B
MGCs: Müller glial cells
mTOR: mammalian target of Rapamycin
MTT: 3-[4,5-dimethylthiazol-2-yl]-2,5 diphenyl tetrazolium bromide
**O**_**2**_: Oxygen
OIR: Oxygen-induced retinopathy
p62: sequestosome 1 (SQSTM 1)
PDR: proliferative diabetic retinopathy
PEDF: pigment epithelium-derived factor
VEGF: Vascular endothelial growth factor
