# Supplementary figures and images for: Rapamycin and Resveratrol Modulate the Gliotic and Pro-Angiogenic Response in Müller Glial Cells Under Hypoxia
Source: Front Cell Dev Biol. 2022 Mar 1;10:855178. doi: 10.3389/fcell.2022.855178 (PMC8921868; doi:10.3389/fcell.2022.855178)

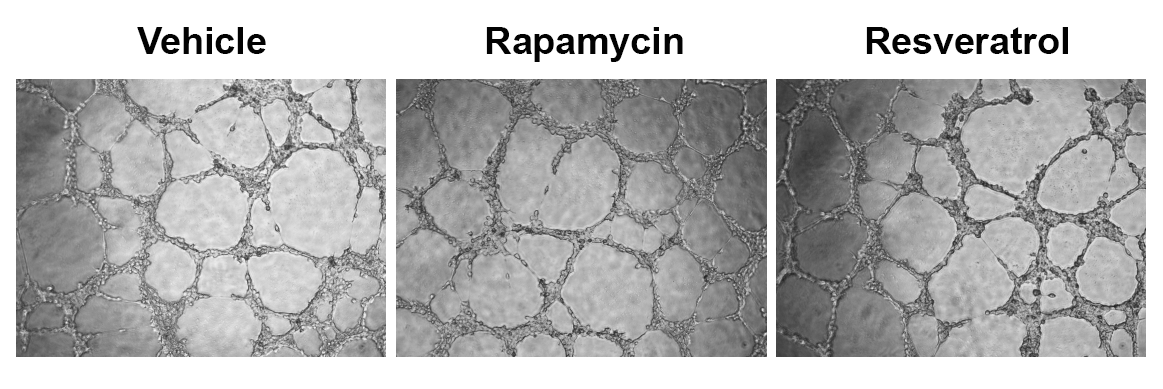

Supplement: Supplementary file 1 [file Image3.TIF]

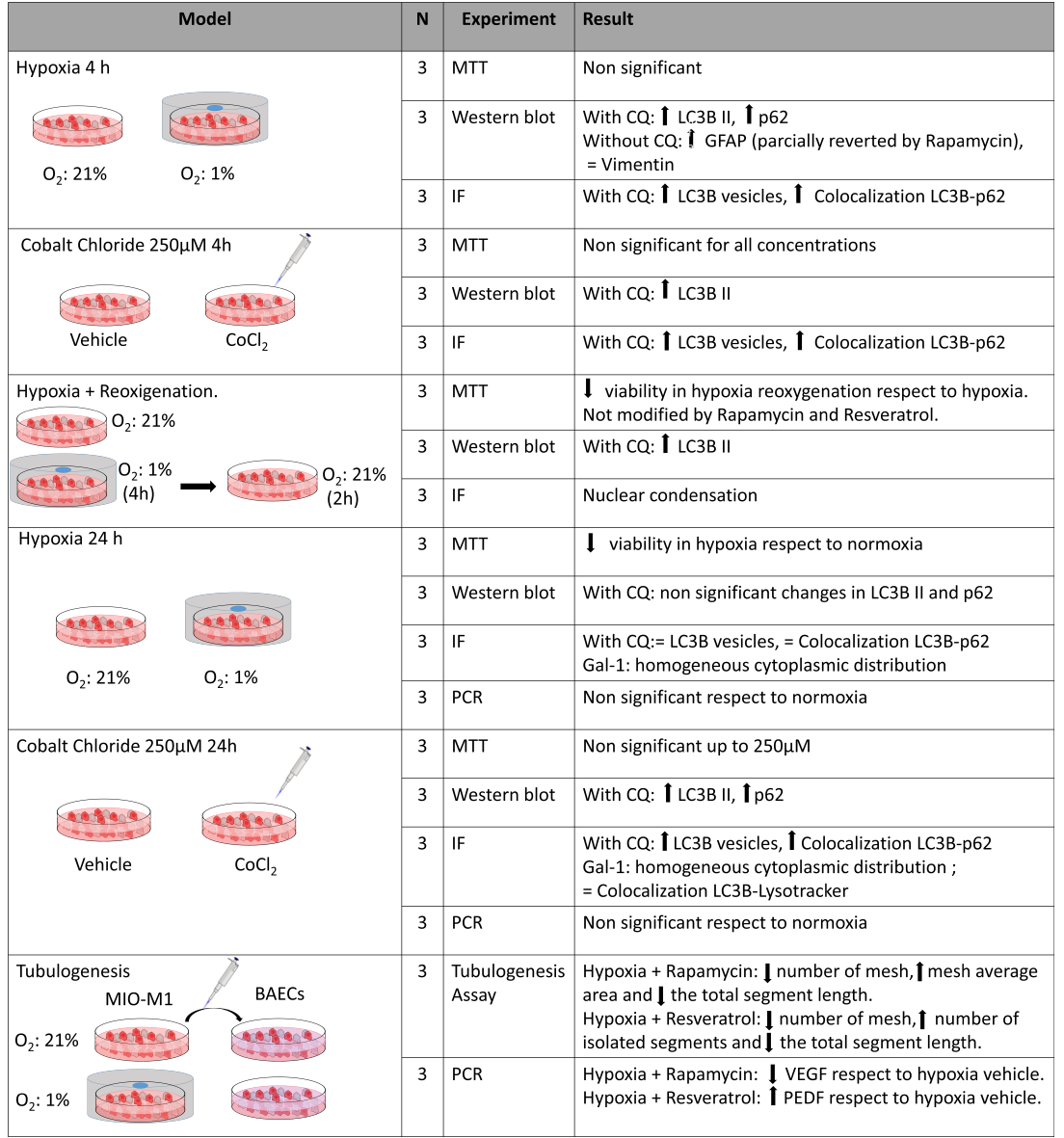

Supplement: Supplementary file 2 [file Image4.TIF]

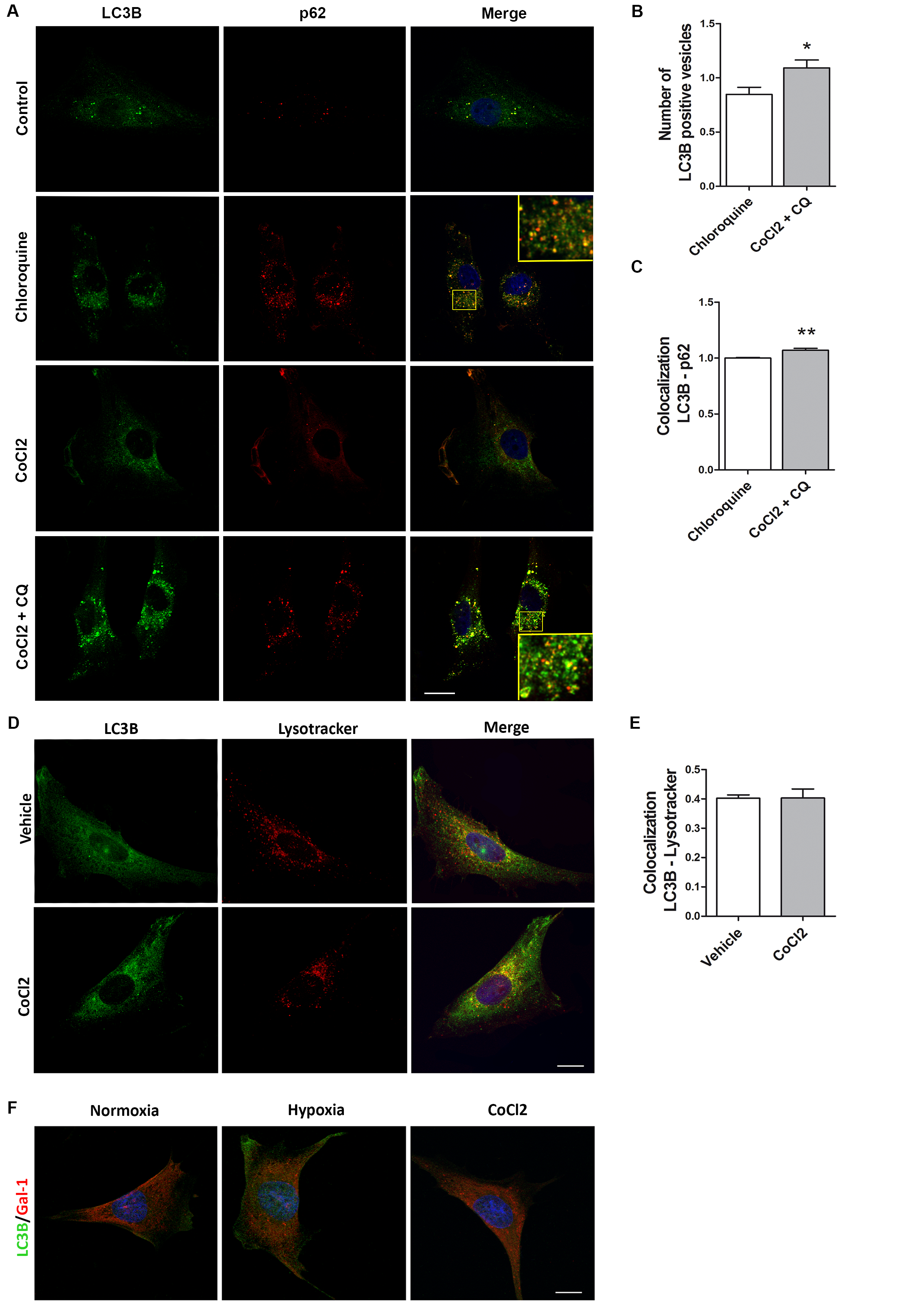

Supplement: Supplementary file 3 [file Image2.TIF]

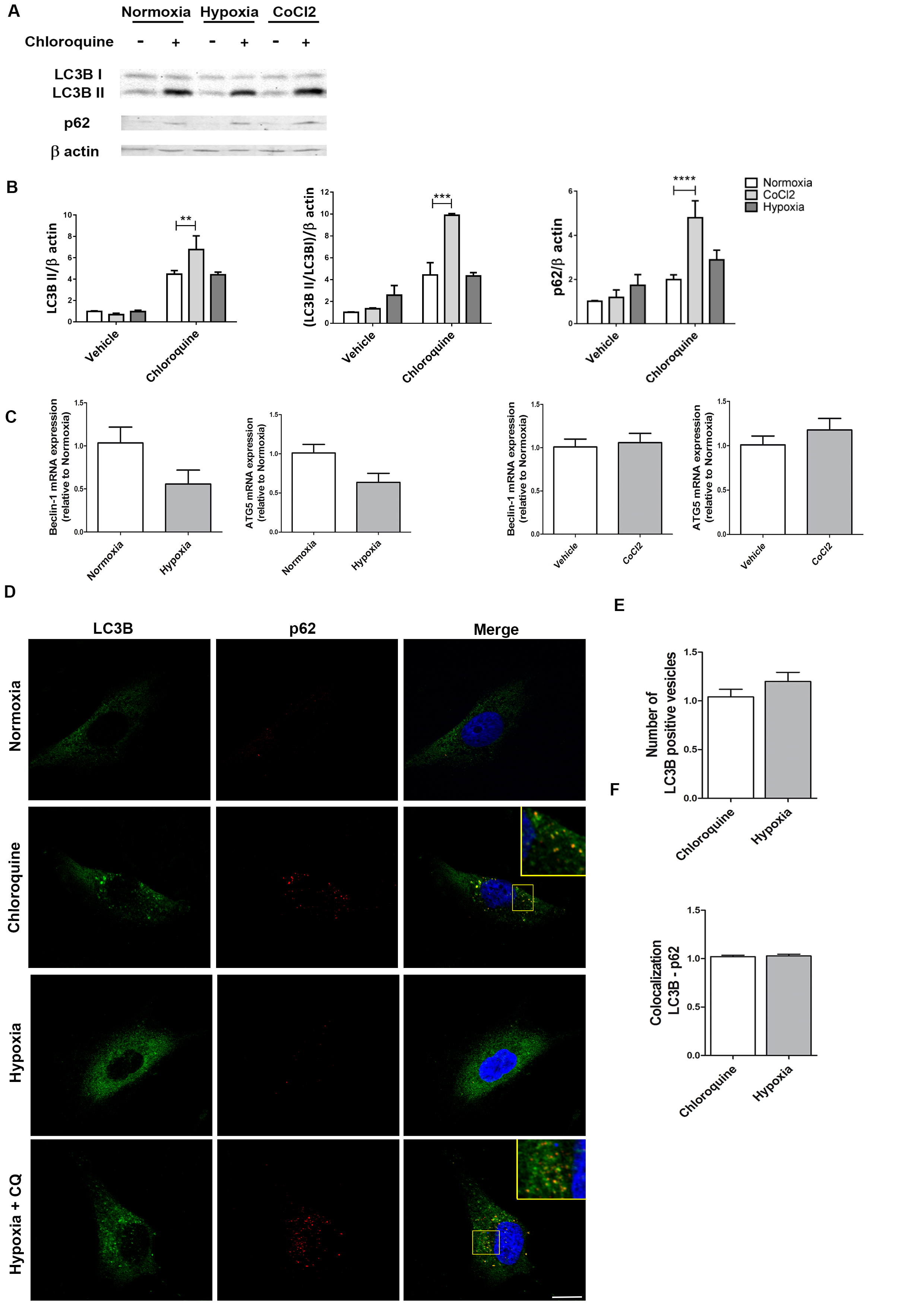

Supplement: Supplementary file 4 [file Image1.TIF]
